# The CXCL5/CXCR2 Axis Attenuates Ferroptosis in Intrahepatic Cholangiocarcinoma Through the Positive Feedback Loop Between PTGS2 Transcriptional Activation and Neutrophil Recruitment

**DOI:** 10.1002/advs.77185

**Published:** 2026-08-17

**Authors:** Chanqi Ye, Kefeng Lin, Shuyao Yang, Yan Zhang, Wenli Gao, Jiaqi Chen, Ying Dong, Rui Bai, Yang Tian, Minghao Wang, Zhijun Yuan, Yuxiao Ma, Yuqin He, Ruyin Chen, Wenguang Fu, Qi Jiang, Qiong Li, Jian Ruan

**Affiliations:** ^1^ Department of Medical Oncology The First Affiliated Hospital, Zhejiang University, School of Medicine and Key Laboratory of Cancer Prevention and Intervention, Ministry of Education Hangzhou Zhejiang People's Republic of China; ^2^ Department of Medical Oncology Cancer Institute Key Laboratory of Cancer Prevention and Intervention Ministry of Education The Second Affiliated Hospital of Zhejiang University, School of Medicine Hangzhou Zhejiang People's Republic of China; ^3^ Department of Medical Oncology Nanyang Second General Hospital Nanyang Henan People's Republic of China; ^4^ Department of Laboratory Medicine Institute of Traditional Chinese Medicine of Sichuan Academy of Chinese Medicine Sciences Chengdu Sichuan People's Republic of China; ^5^ Department of Hepatobiliary and Pancreatic Surgery The Second Affiliated Hospital of Zhejiang University, School of Medicine Hangzhou People's Republic of China; ^6^ Department of Pathology and Pathophysiology Zhejiang University School of Medicine Hangzhou Zhejiang People's Republic of China; ^7^ Department of General Surgery Shizhu Hospital of Traditional Chinese Medicine Chongqing People's Republic of China; ^8^ Department of Hepatobiliary Surgery The Affiliated Hospital of Southwest Medical University Luzhou Sichuan People's Republic of China

**Keywords:** CXCL5/CXCR2, ferroptosis, intrahepatic cholangiocarcinoma, neutrophils recruitment, PTGS2

## Abstract

Intrahepatic cholangiocarcinoma (ICC) presents significant therapeutic challenges due to its late‐stage diagnosis and inherent treatment resistance, resulting in dismal prognosis. While ferroptosis induction has emerged as a promising antitumor strategy, its clinical implementation in ICC has been impeded by poorly defined resistance mechanisms. Here, we systematically investigated the immunomodulatory role of the CXCL5/CXCR2 axis in governing ferroptosis susceptibility through dual mechanistical pathways. In vitro studies revealed that CXCL5/CXCR2 signaling confers ferroptosis resistance by transcriptional upregulation of prostaglandin‐endoperoxide synthase 2 (PTGS2), which can serve as a functional regulatory factor for ferroptosis. Utilizing a tumor‐immune co‐culture platform, we further demonstrated that CXCL5/CXCR2 axis‐driven recruitment of N2 tumor‐associated neutrophils (TANs) orchestrates a protective microenvironment against ferroptosis. Intriguingly, PTGS2 enhances N2 TANs chemotaxis via CCL2 and CCL7, and the recruited N2 TANs subsequently activate the NF‐κB/PTGS2 pathway, thereby establishing a synergistic positive feedback loop. Notably, combinatorial treatment with the CXCR2 antagonist SB225002 and ferroptosis inducer Erastin exhibited synergistic antitumor activity in preclinical ICC models. This study provides mechanistic insights into the ferroptosis‐immune crosstalk and proposes a novel combinatorial therapeutic paradigm for overcoming treatment resistance in ICC.

## Graphical abstract

The CXCL5/CXCR2 axis directly inhibits ferroptosis in ICC cells by activating the NF‐κB/PTGS2 pathway. Meanwhile, the CXCL5/CXCR2 axis recruits N2 TANs, and the activated PTGS2 promotes the secretion of TNF‐α from the recruited N2 TANs via the CCL2/CCL7/CCR2 pathway, thereby activating the NF‐κB/PTGS2 axis and forming a positive feedback loop, which further inhibits ferroptosis in ICC cells.

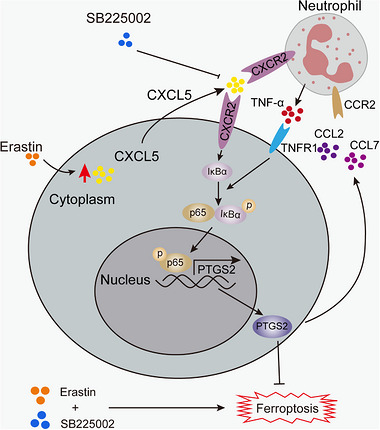

**Abbreviations**
AI: Artificial intelligence
CCK‐8: Cell Counting Kit‐8
ChIP: Chromatin immunoprecipitation
DEG: Differential gene expression
GSEA: Gene set enrichment analysis
H&E: Hematoxylin and Eosin
HIBEC: Human intrahepatic bile duct epithelial cell
ICC: Intrahepatic cholangiocarcinoma
IFN‐γ: Interferon‐γ
IHC: Immunohistochemical
ML: Machine learning
NK cells: natural killer cells
RCD: Regulated cell death
ROC: Receiver operating characteristic
ROS: Reactive oxygen species
RT‐qPCR: Quantitative Real‐Time polymerase chain reaction
scRNA‐seq: Single‐cell RNA sequencing
TANs: Tumor‐associated neutrophils
TEM: Transmission electron microscopy
TME: Tumor microenvironment

## 1 Introduction

Intrahepatic cholangiocarcinoma (ICC) is characterized by an aggressive biological behavior and dismal prognosis [1]. The disease is frequently diagnosed at advanced stages, posing significant clinical challenges and substantial threats to patient survival [2]. Current diagnostic modalities exhibit limited sensitivity and specificity for early‐stage [3]. Therapeutic strategies predominantly encompass surgical resection, radiotherapy, and systemic chemotherapy [4]. However, radiotherapy and chemotherapy are associated with substantial adverse effects and suboptimal therapeutic efficacy. Consequently, there is an imperative need to explore novel approaches, particularly through the investigation of molecular biomarkers and the implementation of precision medicine strategies, to advance clinical decision‐making by improving detection precision, tailoring therapeutic interventions, and ultimately enhancing survival rates among ICC patients.

In the context of ICC, regulated cell death (RCD) modalities have demonstrated significant anti‐tumor efficacy [5]. These RCD‐associated pathways effectively diminish tumor cell viability and enhance therapeutic outcomes [6]. Among emerging RCD paradigms, ferroptosis assumes critical importance in oncology as it may circumvent resistance to conventional therapies [7]. Extensive research has elucidated the molecular underpinnings of ferroptosis, encompassing dysregulated iron homeostasis, GPX4 suppression, and specific lipid peroxidation cascades [8]. Numerous ferroptosis‐inducing agents are currently undergoing clinical evaluation at various stages [9], with certain small molecule compounds demonstrating potent ferroptosis induction and tumor growth inhibition [10]. In ICC specifically, ferroptosis induction has shown efficacy in suppressing tumor cell proliferation, with compounds such as Erastin and RSL3 demonstrating significant increases in reactive oxygen species (ROS) levels, lipid peroxidation, and subsequent ferroptosis induction [11]. However, targeted ferroptosis therapies face several challenges, including limited drug selectivity, potential tumor cell resistance, suboptimal drug delivery and bioavailability, and tumor heterogeneity [12]. Consequently, elucidating novel mechanisms underlying ICC resistance to ferroptosis inducers is imperative for enhancing therapeutic outcomes, prolonging patient survival, and advancing precision oncology strategies.

Immunological regulation fundamentally impacts malignant transformation and clinical intervention outcomes, exerting anti‐tumor effects through the identification and elimination of malignant cells [13]. In ICC, the composition and functional state of the tumor microenvironment (TME) significantly influence disease progression and treatment outcomes [14]. A reciprocal relationship exists between the TME and RCD, as RCD facilitates the clearance of aberrant cells, stimulates immune activation, and enhances anti‐tumor immunity [15]. Ferroptosis, in particular, may elicit immunogenic cell death, potentially releasing tumor antigens and augmenting immune‐mediated tumor destruction [16]. Concurrently, TME can modulate therapeutic resistance by influencing tumor cell survival mechanisms [17]. This interplay suggests that targeting immune‐related genes to regulate tumor cell sensitivity to RCD inducers represents a promising therapeutic avenue. The CXCL5/CXCR2 signaling axis, crucial for immune regulation and neutrophil recruitment [18], warrants particular attention. CXCL5, a chemokine involved in neutrophil chemotaxis to sites of inflammation or neoplasia [19], mediates neutrophil migration through CXCR2 binding, facilitating acute immune responses [20]. In ICC, the CXCL5/CXCR2 axis assumes particular significance, with CXCL5 overexpression correlating with tumor aggressiveness, proliferative capacity, and clinical prognosis [21]. The involvement of immune cells constitutes a critical aspect of CXCL5/CXCR2 axis functionality in cholangiocarcinoma [22]. Previous studies have identified associations between CXCL5 expression and ferroptosis‐related genes [23]. However, the specific impact and regulatory mechanisms of this axis on tumor cell ferroptosis remain unexplored. Consequently, this study elucidates the mechanistic involvement and functional modulation of the CXCL5‐CXCR2 signaling pathway in ferroptotic processes within ICC.

Our findings demonstrate that inhibition of the CXCL5/CXCR2 axis enhances ICC sensitivity to ferroptosis regulatory factors. Mechanistically, the CXCL5/CXCR2 axis activates PTGS2 transcription through NF‐κB phosphorylation, thereby suppressing ferroptosis in ICC. Additionally, N2 tumor‐associated neutrophils (TANs) recruitment mediated by this axis contributes to ferroptosis inhibition. Single‐cell sequencing analysis validated the utility of CXCR2, CXCL5, p65, and PTGS2 as potential biomarkers. Notably, the combination therapy employing the CXCR2 inhibitor SB225002 with Erastin demonstrated promising therapeutic potential in ICC treatment.

## 2 Methods

### 2.1 Data Collection

Transcriptomic dataset OEZ008243 was obtained from the NODE database (https://www.biosino.org/node). GSE89749 and Single‐cell RNA sequencing dataset GSE138709 was retrieved from the GEO database (https://www.ncbi.nlm.nih.gov/geo).

### 2.2 Differential Gene Expression (DEG) Analysis

Gene expression profiling was executed through implementation of the DESeq2 algorithm (release 1.38.3) for comparative analysis of transcriptional modulation, with significance thresholds set at an adjusted *p*‐value (adj.P) < 0.05 and absolute log2‐fold change (|log2FC|) ≥ 1.

### 2.3 CIBERSORT Analysis

Immune cell composition within ICC specimens was computationally deconvoluted using the CIBERSORT analytical framework. Default parameters (1,000 permutation tests, *p*‐value < 0.05) were applied to calculate the fractional composition of distinct immunocyte population within individual specimens.

### 2.4 Univariate Cox Regression Analysis

Univariable Cox regression analysis was performed on ICC data from the NODE database to identify prognosis‐associated genes. The hazard ratio estimation was performed via the survival package (v3.4‐0) in R (v4.2.1).

### 2.5 Gene Set Enrichment Analysis (GSEA)

Based on RNA‐seq differential analysis results, genes were ranked according to their log2 fold change (log2FC). Analysis was performed using clusterProfiler (v4.14.4) in R.

### 2.6 LASSO Regression Analysis

The optimal penalty coefficient λ (lambda.min) was determined to select genes with non‐zero coefficients for building the risk score model via LASSO regression. The analysis was implemented using the glmnet package (v4.1‐3) in R, with the risk score calculated as the sum of each gene's expression level multiplied by its corresponding coefficient (Risk Score = Σ[gene expression × coefficient]).

### 2.7 scRNA‐seq

scRNA‐seq analysis was performed through publicly available ICC data (GSE138709) retrieved from the GEO database. Cellular clusters were classified according to established lineage‐specific gene signatures, including EPCAM for epithelial cells, PTPRC for immune cells, and CD3E for T cells.

### 2.8 Cell Culture

Two established models of human ICC (RBE and HCCC9810) were acquired from the ATCC repository (Manassas, VA, USA). RBE cultures were propagated in high‐glucose DMEM (Gibco, USA), whereas HCCC9810 proliferated in RPMI 1640 (Gibco, USA). Culture media were enriched with 10% FBS (Gibco, USA) and 1% antibiotic‐antimycotic solution (Gibco, USA). Cellular maintenance occurred at physiological conditions (37°C, 5% CO2).

### 2.9 Co‐Culture Model

ICC cells were added to the lower part of the chamber. After 24 h of incubation, the induced N2 TANs were added to the upper chamber. SB225002 (400 nm) was added to the lower medium. Incubate the cells for 7 to 14 days for colony formation assay. For migration of N2 TANs and ELISA, PTGS2‐knockdown ICC cells were implanted into the lower chamber. N2 TANs were incubated into the upper chamber, incubated for 48 h.

### 2.10 Clustered Regularly Interspaced Short Palindromic Repeats Associated Protein 9 (CRISPR‐Cas9)

PTGS2 knockout in ICC cells was achieved using the CRISPR‐Cas9 system as previously described (Zhang laboratory). Briefly, single guide RNAs (sgRNAs) targeting PTGS2 were designed using an online CRISPR design tool and cloned into the lentiCRISPR v2 backbone (Addgene, MA, USA). For lentivirus production, HEK293T cells were co‐transfected with the sgRNA‐containing plasmid, along with the packaging plasmids pVSVg and psPAX2. Viral particles were harvested and concentrated via ultracentrifugation or polyethylene glycol 6000 precipitation. Target ICC cells were then transduced with the concentrated lentivirus at a multiplicity of infection (MOI) of 5 in the presence of 8 µg/mL polybrene, followed by spinoculation at 4°C and 4500 × g for 30 min. After 48 h, transduced cells were selected with puromycin (2–5 µg/mL; Sigma) for two days. To obtain monoclonal populations, cells were subjected to limiting dilution at a density of 60 cells per 10 mL and seeded into 96‐well plates. Clonal expansion was allowed for seven days, and individual colonies were isolated. Successful PTGS2 knockout was confirmed by RT‐PCR and Sanger sequencing.

### 2.11 Cell Transfection

The pLKO.1 plasmid served as the delivery vehicle for shRNA cassettes designed to induce transcript depletion in ICC cell cultures. The shRNA sequences are provided in the Supplementary Table. Lentiviral vectors and plasmids were transfected into 293T cells using Lipofectamine 3000 (Invitrogen, USA) to generate lentiviral particles.

### 2.12 Cell Counting Kit‐8 (CCK‐8) Assay

Cell proliferation was assessed using the CCK‐8 assay according to the protocol (YEASEN, China). Absorbance was measured at 450 nm using a Thermo Scientific spectrophotometer (1510‐00712).

### 2.13 Colony Formation Assay

To quantify proliferative capacity, a density of 1 × 10^3^ cells was inoculated into each well of 6‐well plates and maintained for 7–14 days until distinct colonies emerged. Following methanol fixation, colonies were counterstained with 0.1% crystal violet (Beyotime Biotechnology, China) to facilitate morphological analysis.

### 2.14 Apoptosis Assay

Apoptotic evaluation was performed by plating cells in 6‐well culture dishes and maintaining them until achieving optimal cellular density. Annexin V and propidium iodide (PI) (Beyotime Biotechnology, China) were added at 5 µL each, and the mixture was incubated at room temperature in the dark for 15 min. Apoptotic cells were quantified using flow cytometry within 1 h of staining.

### 2.15 RT‐qPCR

Cellular RNA isolation was performed employing TRIzol reagent (Invitrogen, USA) following the manufacturer's protocol. The extracted RNA was reverse‐transcribed into single‐stranded cDNA using the PrimeScript RT kit (Takara, Japan). The cDNA was diluted and subjected to RT‐qPCR amplification using SYBR Green Master Mix (YEASEN, Shanghai, China). Relative mRNA expression levels were normalized to GAPDH and calculated using the 2−ΔCt method. Primer sequences used in this study are listed in the Supplementary Table.

### 2.16 Western Blot Analysis

Total protein was extracted from cells using cell lysis buffer (CST, USA) supplemented with protease inhibitor (Sigma, Germany). The protein concentration measurements were conducted with a commercial BCA assay (Epizyme, China) according to the manufacturer's specifications. Following SDS‐PAGE separation, protein samples were immobilized on PVDF transfer membranes (Millipore, IPFL00010) via wet transfer methodology for a duration of 90 min. Non‐specific binding sites were passivated using 5% milk protein (Beyotime Biotechnology, China), followed by an extended incubation at 4°C with primary antibodies against CXCL5, PTGS2, P65, BAX, BCL2, MMP9, ARG1, PD‐L1, and GAPDH. Target proteins were visualized through chemiluminescent detection with a commercial ECL system (Epizyme, China).

### 2.17 Immunofluorescence

Glass slides were placed in 12‐well culture dishes prior to cell plating. After treatment, cells were fixed with 4% paraformaldehyde for 30 min at room temperature and permeabilized with 0.1% Triton X‐100 (CST, USA). Non‐specific binding was blocked with 5% bovine serum albumin (BSA) (CST, USA) for 30 min. Cells were incubated with primary antibodies overnight at 4°C, followed by Alexa Fluor 488 or Alexa Fluor 594 (Invitrogen, USA) for 1 h in the dark. Nuclei were counterstained with DAPI (Beyotime Biotechnology, China). Images were acquired using a confocal fluorescence microscope.

### 2.18 ROS Measurement

Cells were seeded in 6‐well plates and treated with ferroptosis inducers (Erastin or RSL3). After treatment, cells were incubated with 5 µm BODIPY‐581/591 C11 (Thermo Fisher Scientific, USA) according to the protocol. Flow cytometric analysis (CytoFLEX, Beckman Coulter) was conducted to determine ROS production levels.

### 2.19 Intracellular Iron Measurement

Total intracellular iron levels were measured using an iron colorimetric detection kit (Elabscience, China) according to the protocol. Absorbance was measured at 593 nm using a multifunctional microplate reader (Tecan), and iron concentration was calculated.

### 2.20 Glutathione (GSH) Assay

Intracellular glutathione levels were measured using a commercial kit (Beyotime Biotechnology, China) to evaluate redox status during ferroptosis. After treatment with Erastin or RSL3, cells were harvested, washed with cold PBS, and lysed in 5% sulfosalicylic acid via freeze–thaw cycles. Lysates were centrifuged at 12 000 × g for 10 min at 4°C, and supernatants were collected. For total GSH, 20 µL of supernatant or standard was mixed with DTNB in a 96‐well plate and incubated at 37°C for 5 min, followed by addition of NADPH and glutathione reductase. Absorbance at 412 nm was recorded every minute for 5 min. For GSSG, an aliquot was pre‐treated with 2‐vinylpyridine before the same procedure. Reduced GSH was calculated as total GSH – 2 × GSSG. Results were normalized to protein content (BCA method) and expressed as nmol/mg protein. All assays were performed in triplicate, and data are shown as mean ± SD.

### 2.21 Luciferase Reporter Assay

The promoter region of PTGS2 was inserted into the pGL4.10 luciferase reporter plasmid (Promega, Madison, WI, USA). The reporter vector was introduced into ICC cells alongside a β‐galactosidase‐encoding plasmid (PSV‐β‐gal) as an internal control. Luciferase activity was measured using a luciferase reporter kit (Promega, USA) and a luminometer (Berthold Tech., Germany). β‐galactosidase activity was quantified using the β‐galactosidase Assay Kit (Promega, USA), and luciferase activity was normalized to β‐gal activity.

### 2.22 Chromatin Immunoprecipitation (ChIP)

ChIP experiments were initiated by fixing cells with 1% formaldehyde (10 min, RT), followed by quenching with 0.2 m glycine (5 min), following the protocol provided by the ChIP assay kit (Beyotime Biotechnology, China). Primer sequences are provided in the Supplementary Table.

### 2.23 Transmission Electron Microscopy (TEM)

Cells were fixed with 2.5% glutaraldehyde in PBS for 2.5 h at room temperature. After washing with PBS, specimens were incubated in 0.1% tannic acid (Millipore‐filtered, cacodylate‐buffered), followed by secondary fixation with 1% OsO_4_ and subsequent uranyl acetate staining. Samples were then dehydrated and embedded in Spurr's resin. Ultrathin sections were prepared and imaged using a transmission electron microscope.

### 2.24 Animal Models of ICC and Subcutaneous Tumor

All procedures involving animals were performed following protocols authorized by the Institutional Animal Care and Use Committee (IACUC) of ZJCL (ZJCLA‐IACUC‐20010826). Female C57BL/6 mice aged 5–7 weeks were housed under specific pathogen‐free (SPF) conditions with ad libitum access to food and water. Prior to experimentation, all animals were assessed for health and acclimated to the environment. Spontaneous in situ ICC was induced via hydrodynamic tail vein injection (HTVI) of a plasmid mixture containing T3‐EF1a‐NICD, PT3‐myr‐AKT‐HA, and SB100. One week post‐injection, mice were administered experimental treatments. On day 28, tumor tissues were harvested for subsequent analyses. Female BALB/c nude mice aged 4–6 weeks were housed under SPF conditions with ad libitum access to food and water. Each group was subcutaneously injected with 100 µL of the cell Matrigel matrix gel suspension under the armpit, with the cell concentration being 1×10^6^ cells/100 µL. Tumor size of the mice was monitored every 2 days, and the tumor volume was calculated. At the end of the experiment, the mice were sacrificed, the tumor tissues were stripped, weighed, and photographed.

### 2.25 Isolation and Polarization of Neutrophils

Fresh peripheral whole blood was collected from healthy volunteers. The study obtained written informed consent from the volunteers and was approved by the Clinical Research Ethics Committee of the First Affiliated Hospital, Zhejiang University. Neutrophils were isolated from peripheral blood using Polymorphprep (Serumwerk Bernburg AG, Germany). 5 mL of Polymorphprep was added to a 15 mL centrifuge tube, followed by the gentle addition of 5 mL of undiluted whole blood containing EDTA anticoagulant to maintain the layering. Then, at 18°C–22°C, a 500 g centrifuge was used for 30 min, and the centrifuge speed was gradually reduced at the end of the centrifugation. The layering could be observed in the tube, and the middle layer band was the neutrophils. The neutrophils were aspirated and transferred to a new centrifuge tube, and immediately balanced the osmotic pressure with an equal volume of 0.5 times concentration of the culture medium. A 400 g centrifuge was used for 10 min, the supernatant was discarded, and erythrocyte lysis buffer (Solarbio, China) was added to lyse the residual red blood cells. Another 400 g centrifuge was used for 10 min, the supernatant was discarded. The precipitated cells were resuspended in the culture medium and stored for subsequent experiments. The isolated primary neutrophils were cultured in RPMI 1640 complete medium containing 3 µm QVD‐OPh as the apoptosis inhibitor. The complete medium was supplemented with polarizing inducers at final concentrations of 25 mm L‐lactic acid, 10 µm adenosine, 20 ng/mL TGF‐β1, 10 ng/mL IL‐10, 20 ng/mL PGE2, and 100 ng/mL G‐CSF. The cultures were conducted for 24 or 48 h to establish the N2 polarizing induction group.

### 2.26 Enzyme‐Linked Immunosorbent Assay (ELISA)

Cell culture supernatant was collected and centrifuged at 4°C for 10 min at 3000 rpm. Add the standard sample and the test supernatant to the pre‐coated 96‐well plate separately. Each group has three duplicate wells. The plate was incubated for 2 h and washed 5 times with PBST. Add the detection primary antibody and incubate for 1 h. Wash the plate 5 times. Then add the secondary antibody conjugated with HRP, incubate in the dark for 30 min, and wash the plate 5 times with PBST. Add the TMB color development substrate to each well and incubate at room temperature in the dark for 15 min. Finally, add the stop solution and immediately measure the OD value at 450 nm. Fit a standard curve based on the OD values of the series of standard samples, and calculate the protein concentration of each sample accordingly.

### 2.27 Hematoxylin and Eosin (H&E) Staining and Immunohistochemical (IHC) Analysis

For H&E staining, sections were incubated with hematoxylin (Beyotime Biotechnology, China) for 3–10 min at room temperature, followed by eosin staining for 30 s. Slides were dehydrated, cleared in xylene, and mounted with neutral resin. For IHC staining, antigen retrieval was performed by boiling sections in citric acid buffer (Beyotime Biotechnology, China) for 10 min. To eliminate endogenous peroxidase reactivity, samples were treated with 3% H_2_O_2_ (10 min incubation). Tissue sections were first treated with 5% goat serum (30 min blocking step) before overnight incubation (4°C) with primary antibodies targeting Ki67, Ly6G, and GPX4. Following washes, tissue sections were exposed to HRP‐linked secondary antibodies (Proteintech, China) for 30 min under ambient conditions. Signal was colored via 3,3′‐diaminobenzidine (DAB), and nuclei were counterstained with hematoxylin. Slides were dehydrated, cleared in xylene, and mounted with neutral resin. All the slices were randomly numbered and independently scored by two researchers who were unaware of the group assignments. The percentage of positive cells was rated per high‐power field by 200× magnification as follows: 0 for sections with 1% positive cells; 1 for 1%–25%; 2 for 26%–50%; 3 for 51%–75%; 4 for 76%–100%. The staining intensity was graded as follows: weak intensity graded as 1, moderate intensity as 2, and high intensity as 3. The final point is the percentage of positive cells multiplied by staining intensity. Negative expression was scored 0–1, weak expression was scored 2–4, and strong expression was scored 6–12. If there is a significant difference in the scores given by the two researchers, they should jointly review the slides in a blind manner to reach a consensus on the scores.

### 2.28 Statistical Analysis

Statistical computations were carried out with GraphPad Prism 8.0 and R statistical package (v4.2.1). Survival curves were generated using the Kaplan‐Meier method, and between‐group differences were assessed using log‐rank tests. Two‐way analysis of variance (ANOVA) was employed to evaluate interaction effects between independent variables on continuous outcomes. Comparisons between two groups were performed using independent two‐sample Student's t‐tests. Two multiple testing correction strategies were used to control Type I error: the Benjamini–Hochberg FDR procedure for large‐scale multi‐indicator comparisons, and Bonferroni adjustment for a small set of pre‐defined pairwise contrasts. All displayed significance levels were calculated from corrected statistical outputs, with thresholds set at FDR < 0.05 (Benjamini–Hochberg) or adjusted *p* < 0.05 (Bonferroni). Receiver operating characteristic (ROC) curve analysis was conducted to assess the predictive performance of prognostic models, with area under the curve (AUC) and 95% CI reported. For comparisons across multiple groups, one‐way ANOVA was employed, supplemented by Tukey's post hoc analysis (statistical significance defined as *p* < 0.05).

## 3 Results

### 3.1 CXCL5/CXCR2 Axis Regulates Ferroptosis and Malignant Progression in ICC

**FIGURE 1:**
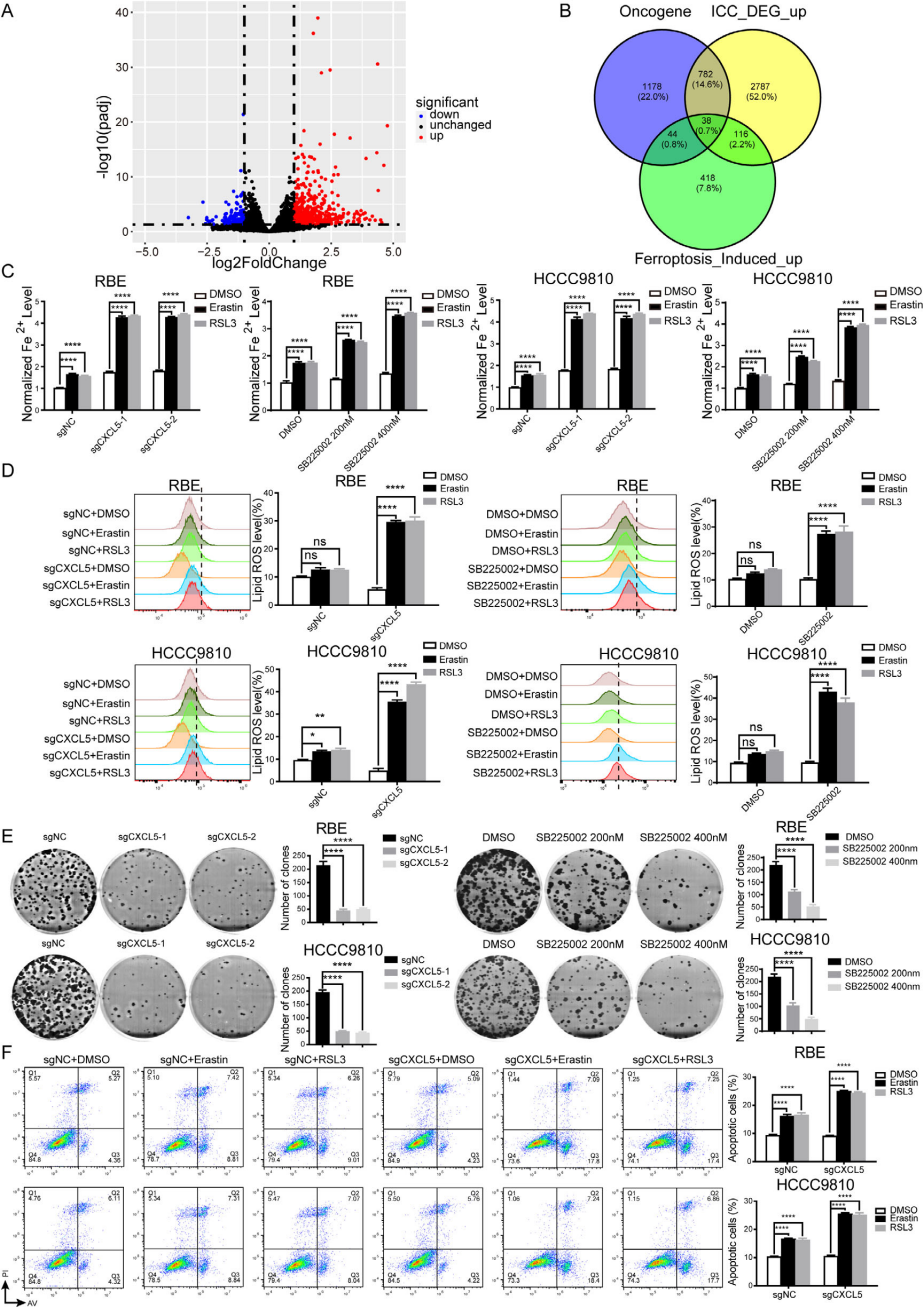
CXCL5/CXCR2 axis regulates the ferroptosis and malignant progression of ICC. (A) Volcano plots illustrate DEGs between DMSO and Erastin groups in HCCC9810 cells. Values are presented as the log2 of foldchange. (B) Venn diagram shows the overlapping among oncogenes, up‐regulated genes of ICC and ferroptosis‐induced upregulated genes. (C) The Fe^2+^ assays in HCCC9810 and RBE cell lines treating with low concentrations of Erastin (2 µm) and RSL3 (1 µm) in CXCL5 knockout or CXCR2 inhibition groups respectively. Data are means ± S.D., n = 3. (D) The lipid ROS assays in HCCC9810 and RBE cell lines treating with low concentrations of Erastin (2 µm) and RSL3 (1 µm) in CXCL5 knockout or CXCR2 inhibition groups respectively. Data are means ± S.D., n = 3. (E) Colony formation assays in the HCCC9810 and RBE cell lines when knocking out CXCL5 or inhibiting CXCR2 respectively. Data are means ± S.D., n = 3. (F) The apoptosis assays in the HCCC9810 and RBE cell lines treated with low concentrations of Erastin (2 µm) and RSL3 (1 µM) when knocking out CXCL5. Data are means ± S.D., n = 3. ^*^ *p* < 0.05, ^**^ *p* < 0.01, ^***^ *p* < 0.001, ^****^ *p* < 0.0001.

To investigate the role of immune‐related genes in ICC ferroptosis, we induced ferroptosis in HCCC9810 cells by using Erastin and performed RNA‐seq to discover DEGs. Analysis revealed 616 upregulated and 205 downregulated genes (Figure 1A). Given that upregulated genes following ferroptosis induction may contribute to ferroptosis resistance, we focused on 38 upregulated genes highly expressed in ICC samples and related to poor prognosis according to NODE database (OEZ008243) (|log2FC| ≥ 1, HR > 1, *p* < 0.05) (Figure 1B). Among these, CXCL3 and CXCL5, both closely associated with TME, were selected for further investigation due to their potential role in modulating ferroptosis through immune pathways. To investigate the function of CXCL3 and CXCL5 in ferroptosis regulation, we detected the IC50 values of Erastin and RSL3 in control, CXCL3‐knockdown, and CXCL5‐knockdown groups. Results demonstrated that CXCL5 knockdown significantly reduced the IC50 values of both ferroptosis inducers, whereas CXCL3 knockdown had no significant effect (Figure S1A). These findings suggest that CXCL5 plays a critical role in regulating ICC ferroptosis. Then, we assessed the protein expression levels of CXCL5 in ICC cell lines and human intrahepatic bile duct epithelial cells (HIBEC). Our results identified that HCCC9810 and RBE are suitable for subsequent experiments (Figure S1B). To further validate the above hypothesis, we treated ICC cells with low concentrations of Erastin (2 µm) and RSL3 (1 µm) following CXCL5 knockout/knockdown or CXCR2 inhibition and measured intracellular Fe^2+^ concentrations and ROS levels. CXCL5/CXCR2 inhibition group have significantly higher Fe^2+^ concentrations and lipid ROS levels compared to the control group, indicating that CXCL5/CXCR2 axis desensitizes ICC cells to ferroptosis inducers (Figure 1C,D and Figure S1D,E). Additionally, our results demonstrated that CXCL5 knockout/knockdown or CXCR2 inhibition significantly suppresses ICC cell proliferation and promotes apoptosis (Figure 1E,F and Figure S1F,G). And apoptosis is a downstream secondary effect of ferroptosis, rather than an independent signaling pathway mediated by CXCL5/CXCR2 (Figure S1H). Collectively, these results demonstrate that inhibiting CXCL5/CXCR2 axis promotes ferroptosis and inhibits malignant progression in ICC.

### 3.2 The CXCL5/CXCR2 Axis Regulates ICC Ferroptosis via PTGS2

**FIGURE 2:**
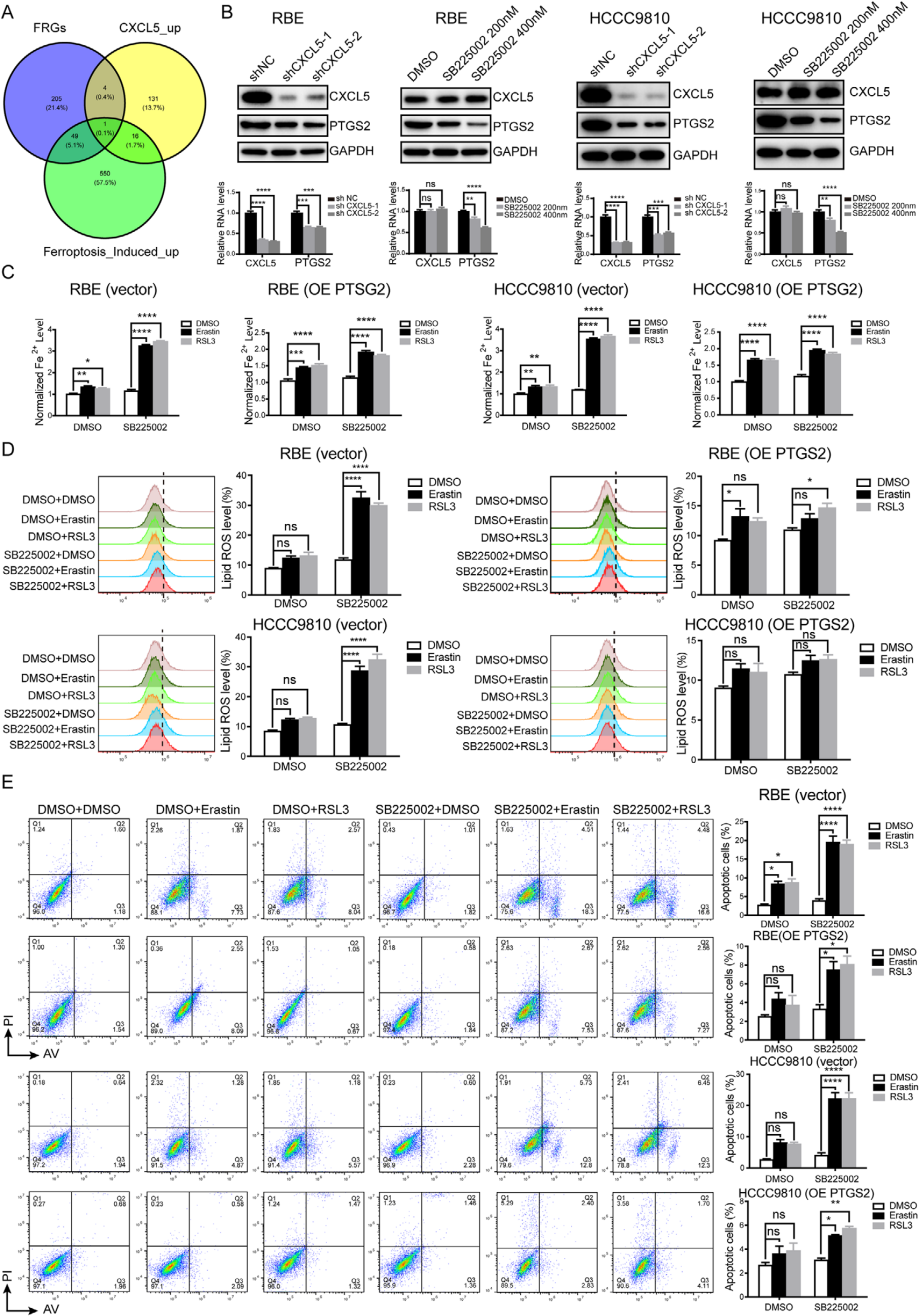
CXCL5/CXCR2 axis regulates ICC ferroptosis through PTGS2. (A) Venn diagram shows the overlapping among ferroptosis‐related genes, highly expressed genes in CXCL5 high and low expression group and ferroptosis‐induced upregulated genes. (B) Western blot analysis and RT‐qPCR assay of CXCL5 and PTGS2 protein and mRNA expressions in HCCC9810 and RBE cell lines with the treatment of knocking down CXCL5 or using SB225002. Data are means ± S.D., n = 3. (C) The Fe^2+^ assays in RBE and HCCC9810 cell lines treating with low concentrations of Erastin (2 µm) and RSL3 (1 µm) when inhibit CXCR2 by SB225002 and overexpress PTGS2. Data are means ± S.D., n = 3. (D) The lipid ROS assays in CXCR2 inhibition by SB225002 and overexpressing PTGS2 ICC cell lines when treated with low concentrations of Erastin (2 µm) and RSL3 (1 µm). Data are means ± S.D., n = 3. (E) The apoptosis assays in CXCR2 inhibition by SB225002 and overexpressing PTGS2 ICC cell lines when treated with low concentrations of Erastin (2 µm) and RSL3 (1 µm). Data are means ± S.D., n = 3. ^*^ *p* < 0.05, ^**^ *p* < 0.01, ^***^ *p* < 0.001, ^****^ *p* < 0.0001.

To elucidate the molecular mechanisms underlying CXCL5/CXCR2‐mediated ferroptosis regulation, we stratified 255 ICC samples from the NODE database (OEZ008243) into high‐ and low‐CXCL5 expression groups and performed DEG analysis (Figure S1C). Among the upregulated genes, PTGS2, a known ferroptosis‐related gene, was significantly elevated following Erastin treatment (Figure 2A). We hypothesized that the CXCL5/CXCR2 axis regulates ferroptosis through PTGS2. To test this, we measured PTGS2 protein and mRNA levels after CXCL5 knockdown or CXCR2 inhibition. Both interventions significantly reduced PTGS2 expression (Figure 2B). Furthermore, knockdown of PTGS2 significantly decreased the IC50 values of Erastin and RSL3 in ICC cells, confirming its role in ferroptosis regulation (Figure S2A,B). Treatment with low concentrations of Erastin (2 µm) and RSL3 (1 µm) following PTGS2 knockdown resulted in increased Fe^2+^ levels and lipid ROS, further supporting PTGS2's involvement in ferroptosis (Figure S2C,D). Treatment with low concentrations of Erastin (2 µm) and RSL3 (1 µm) following PTGS2 knockdown resulted in increased GSSG/GSH levels, indicating that PTGS2 knockdown may consume GSH to regulate ferroptosis (Figure S2E). Rescue experiments demonstrated that CXCL5 knockdown increased ICC sensitivity to ferroptosis inducers in control groups but had no significant effect in PTGS2‐overexpressing cells (Figure S2F,G). To eliminate the off‐target effect caused by the knockdown of CXCL5, we conducted another rescue experiment using SB225002. The results showed that CXCR2 inhibition also increased ICC sensitivity to ferroptosis inducers in control groups but had no significant effect in PTGS2‐overexpressing cells (Figure 2C 2D,E). These findings indicate that the CXCL5/CXCR2 axis can regulates ICC ferroptosis through PTGS2.

### 3.3 The CXCL5/CXCR2 Axis Activates PTGS2 Transcription via NF‐κB Phosphorylation

**FIGURE 3:**
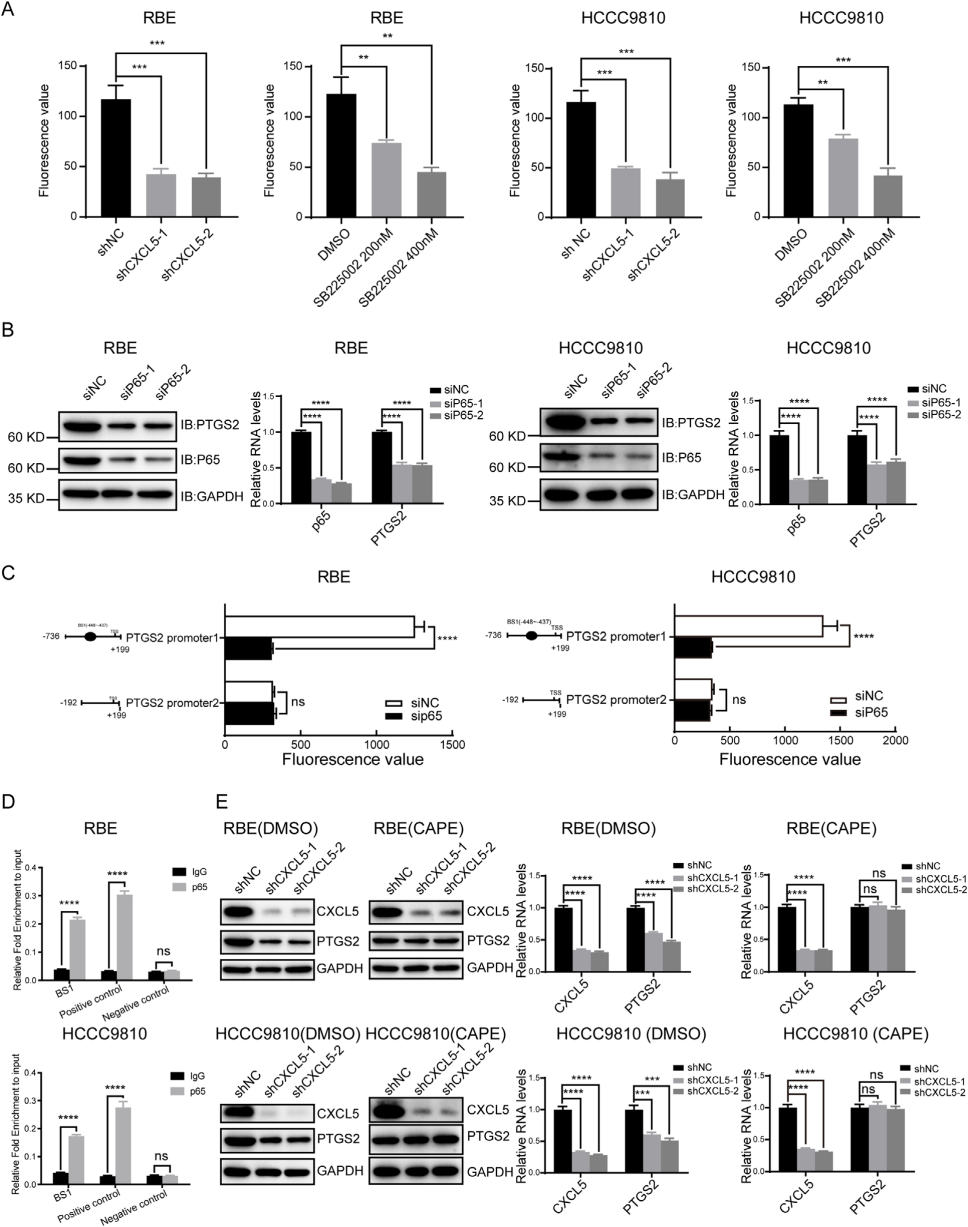
The CXCL5/CXCR2 axis transcripts PTGS2 through activating phosphorylation of NF‐κB. (A) Luciferase activities detected the NF‐κB function after knocking down CXCL5 or using SB225002 in HCCC9810 and RBE cells. Data are means ± S.D., n = 3. (B) Western blot analysis and RT‐qPCR assay of RELA and PTGS2 expressions in HCCC9810 and RBE cell lines with the treatment of knocking down p65. Data are means ± S.D., n = 3. (C) Luciferase activities after truncation fragments in HCCC9810 and RBE cells. Data are means ± S.D., n = 3. (D) ChIP‐qPCR of the PTGS2 promoter, immunoprecipitated with antibodies against p65 in HCCC9810 and RBE cells. Positive control targeting a canonical NF‐κB target site. Negative control targeting a non‐coding genomic region lacking NF‐κB binding motifs. Data are means ± S.D., n = 3. (E) Western blot analysis and RT‐qPCR assay of PTGS2 and CXCL5 expressions in shNC and shCXCL5 HCCC9810 and RBE cell lines with the treatment of DMSO or CAPE. Data are means ± S.D., n = 3. Data are means ± S.D., n = 3. ^*^ *p* < 0.05, ^**^ *p* < 0.01, ^***^ *p* < 0.001, ^****^ *p* < 0.0001.

To elucidate the molecular mechanism by which the CXCL5/CXCR2 axis regulates PTGS2, we focused on NF‐κB, a critical transcriptional regulator that bridges immunity and tumorigenesis. Recent evidence suggests that NF‐κB can transcriptionally regulate PTGS2. We hypothesized that the CXCL5/CXCR2 axis modulates PTGS2 expression by activating NF‐κB. To test this hypothesis, we assessed NF‐κB transcriptional activity and p65 nuclear localization following CXCL5 knockdown or CXCR2 inhibition. Both interventions significantly reduced NF‐κB transcriptional activity (Figure 3A). To further confirm NF‐κB's role in PTGS2 regulation, we examined PTGS2 expression levels after NF‐κB knockdown. Results demonstrated that NF‐κB knockdown significantly decreased both PTGS2 protein and mRNA levels, supporting the hypothesis of transcriptional regulation (Figure 3B). To identify the specific NF‐κB binding sites within the PTGS2 promoter, we conducted promoter activity assays and chromatin immunoprecipitation (ChIP) experiments targeting two predicted NF‐κB binding regions (Figure S3). These experiments revealed that NF‐κB binds to the (−448 to −437) site in the PTGS2 promoter, thereby regulating its transcriptional activity (Figure 3C,D). To determine whether the CXCL5/CXCR2 axis activates PTGS2 through NF‐κB, we performed a rescue experiment. CXCL5 knockdown significantly reduced PTGS2 expression in the control group; however, this effect was abolished in the presence of the NF‐κB inhibitor CAPE, indicating that NF‐κB is essential for CXCL5/CXCR2‐mediated PTGS2 activation (Figure 3E). In summary, these findings demonstrate that the CXCL5/CXCR2 axis activates PTGS2 transcription through NF‐κB phosphorylation, providing a mechanistic link between immune signaling and ferroptosis regulation in ICC.

### 3.4 CXCL5 Promotes Malignant Progression and Inhibits Ferroptosis in ICC by Recruiting N2 TANs

The recent reports suggested that CXCL5 plays a pivotal function in N2 TANs recruitment. According to these findings, we hypothesized that CXCL5 modulate ferroptosis in ICC by recruiting N2 TANs. To investigate this, we performed CIBERSORT analysis on ICC data from the NODE database, revealing significantly higher neutrophil infiltration in the CXCL5 high‐expression group compared to the CXCL5 low‐expression group (Figure S4A). This observation supports the hypothesis that CXCL5 facilitates neutrophil recruitment.

**FIGURE 4:**
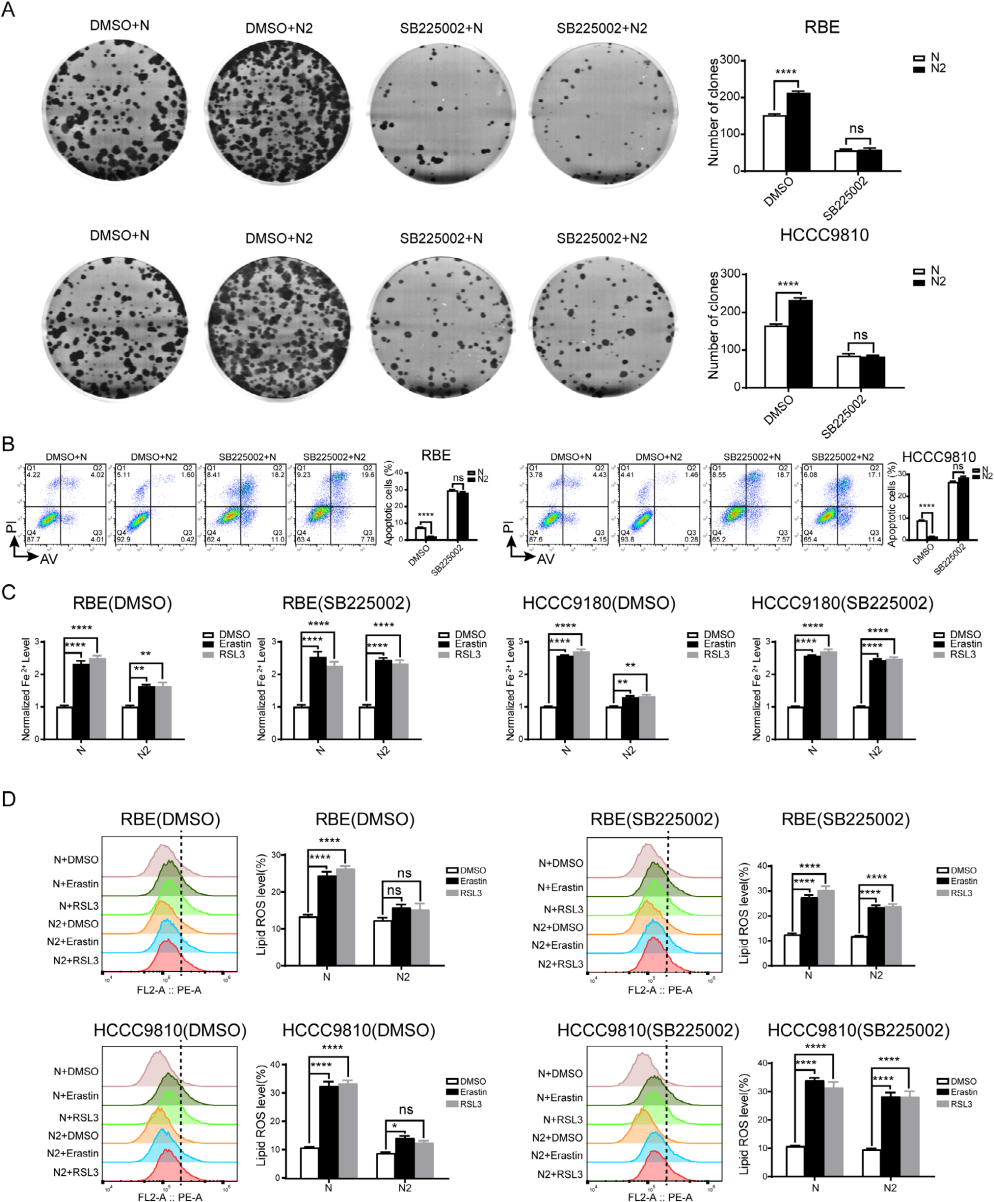
CXCL5 promotes malignant progression of ICC and inhibits ferroptosis by recruiting N2 TANs. (A) Colony formations were performed in DMSO or SB225002 groups when co‐cultured with N2 TANs. Data are means ± S.D., n = 3. (B) Apoptosis assays were performed in DMSO or SB225002 groups when co‐cultured with N2 TANs. Data are means ± S.D., n = 3. (C) The Fe^2+^ assays in DMSO or SB225002 groups when co‐cultured with N2 TANs. Data are means ± S.D., n = 3. (D) The lipid ROS assays in DMSO or SB225002 groups when co‐cultured with N2 TANs. Data are means ± S.D., n = 3. ^*^ *p* < 0.05, ^**^ *p* < 0.01, ^***^ *p* < 0.001, ^****^ *p* < 0.0001.

N2 is a type of neutrophil that promotes tumor growth. After isolating neutrophils from the whole blood of healthy patients, we further induced them to polarize into the N2 type, and verified the success of the induction through western blot assay (Figure S4B). To further investigate whether CXCL5 influences the malignant progression and ferroptosis of ICC through N2 TANs recruitment, we conducted a series of rescue experiments. Results demonstrated that co‐cultured with N2 TANs significantly increase their clonogenic ability. This effect was not significant when ICC cells were treated with SB225002, indicating that CXCL5 promotes ICC proliferation, at least in part, by recruiting N2 TANs (Figure 4A). The apoptosis assay presented the similar result (Figure 4B). Next, we explored the impact of CXCL5‐mediated N2 TANs recruitment on ICC ferroptosis. We cultured ICC cells with N2 TANs in both DMSO group and SB225002 group, followed by treatment with low concentrations of Erastin (2 µm) and RSL3 (1 µm). Intracellular Fe^2+^ levels and lipid ROS were then measured. Co‐cultured ICC cells decreased their sensitivity to ferroptosis inducers in DMSO group, as evidenced by elevated Fe^2+^ and lipid ROS levels, which was not observed in SB225002 group (Figure 4C,D). Previous studies have demonstrated that neutrophils can activate the NF‐κB signaling pathway in tumor cells via TNF‐α. To investigate whether N2 TANs exert a protective effect on ICC by secreting TNF‐α, we conducted an ELISA experiment on the N2 TANs. The results showed an increase in TNF‐α when co‐cultured with ICC cells, thereby confirming that TNF‐α can mediate the protective effect of N2 TANs on ICC (Figure S4C). In summary, these findings demonstrate that CXCL5 drives ICC malignant progression and inhibits ferroptosis through N2 TANs recruitment, highlighting its dual role in promoting tumor growth and resistance to cell death.

### 3.5 Activation of PTGS2 and Recruitment of N2 TANs Form a Positive Feedback Loop

**FIGURE 5:**
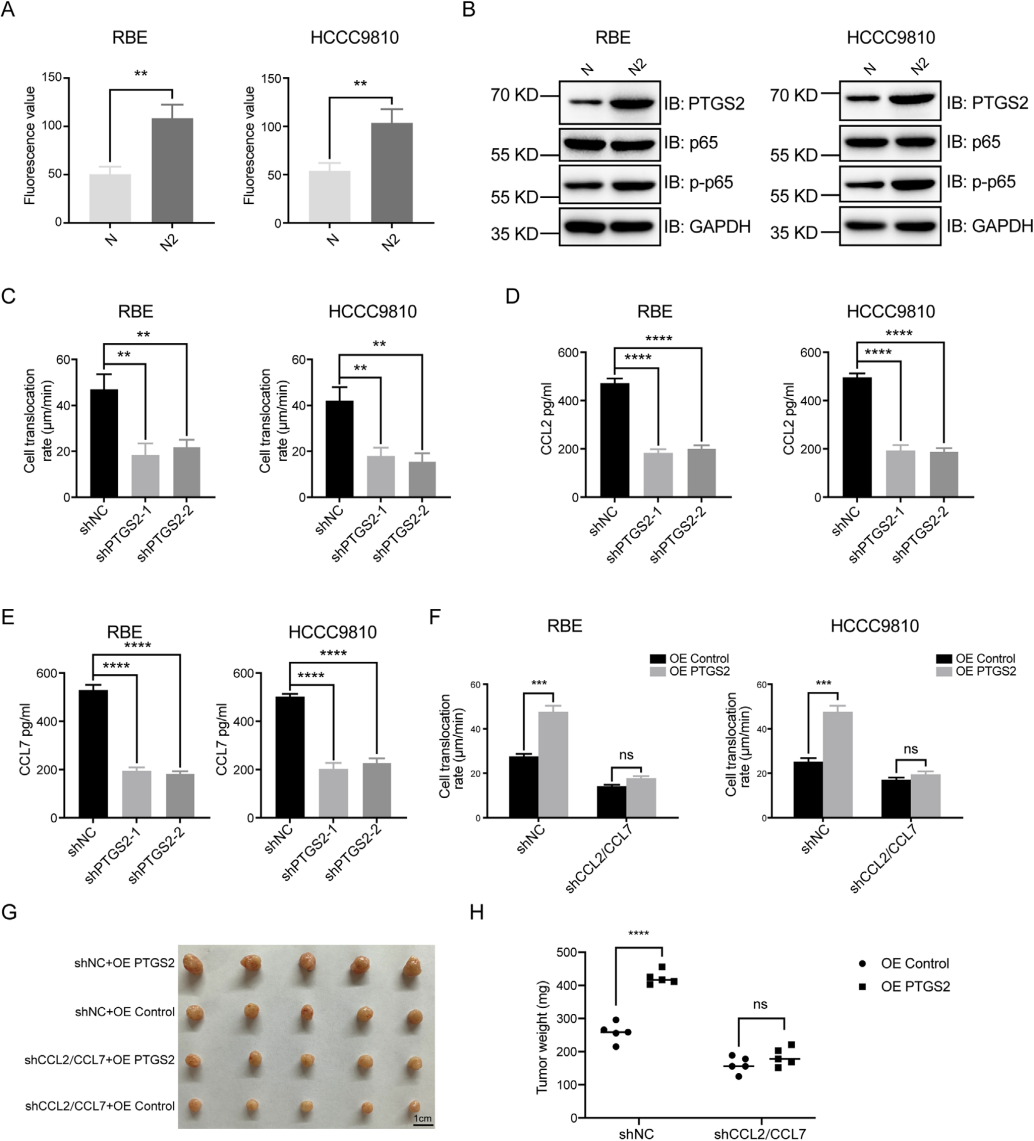
Activation of PTGS2 and Recruitment of N2 TANs Form a Positive Feedback Loop. (A) NF‐κB activity in ICC cells was assessed using a reporter gene assay following co‐culture with N2 TANs. (B) PTGS2/ NF‐κB expression in ICC cells was assessed using western blot assay following co‐culture with N2 TANs. (C) Migration rate of N2 TANs was quantified via cell counting after co‐culture with PTGS2‐knockdown ICC cells in a Transwell chamber system. (D,E) CCL2 and CCL7 concentration was measured by ELISA following co‐culture of PTGS2‐knockdown ICC cells with N2 TANs in a Transwell chamber. (F) Migration rates of N2 TANs were quantified by cell counting after co‐culture with CCL2/CCL7‐knockdown and PTGS2‐overexpressed ICC cells in a Transwell chamber system. Data are means ± S.D., n = 3. (G) Images of subcutaneous tumors in nude mice of different treatment groups. (H) Weight of subcutaneous tumors in nude mice of different treatment groups. Data are means ± S.D., n = 5. ^*^ *p* < 0.05, ^**^ *p* < 0.01, ^***^ *p* < 0.001, ^****^ *p* < 0.0001.

Previous studies have demonstrated that neutrophils can activate the NF‐κB signaling pathway in tumor cells via TNF‐α. Thus, we hypothesized that N2 TANs recruitment might activate the NF‐κB/PTGS2 pathway. To validate this hypothesis, we co‐cultured ICC cells with N2 TANs and measured NF‐κB and PTGS2 expression in ICC cells. The results demonstrated that N2 TANs significantly activated the NF‐κB/PTGS2 pathway (Figure 5A,B). Activated PTGS2 may further promote N2 TANs chemotaxis via the CCL2/CCL7 axis. To verify this mechanism, we co‐cultured PTGS2‐knockdown ICC cells with N2 TANs in a Transwell system and measured N2 TANs migration rates alongside CCL2 and CCL7 concentrations in the medium. The data revealed that PTGS2 knockdown led to significant reductions in N2 TANs migration rates and CCL2/CCL7 levels (Figure  5D,5C,E). We also co‐cultured CCL2/CCL7‐knockdown and PTGS2‐overexpressed ICC cells and N2 TANs in the Transwell system and quantified N2 TANs migration rates. The results showed that overexpressed PTGS2 increased migration rates of N2 TANs, whereas no significant changes were observed in the CCL2/CCL7 knockdown group (Figure 5F). To further validate the regulatory relationship between the PTGS2 and CCL2/CCL7 axis, we conducted in vivo experiments using a nude mouse subcutaneous tumor formation model. The results showed that after knockdown of CCL2/CCL7, the increase in PTGS2 expression was unable to promote tumor growth (Figure 5G,H). Collectively, these findings indicate that PTGS2 activation and N2 TANs recruitment establish a self‐reinforcing positive feedback loop.

### 3.6 SB225002 And Erastin Synergistically Inhibit Malignant Progression and Promote Ferroptosis in ICC Cells

**FIGURE 6:**
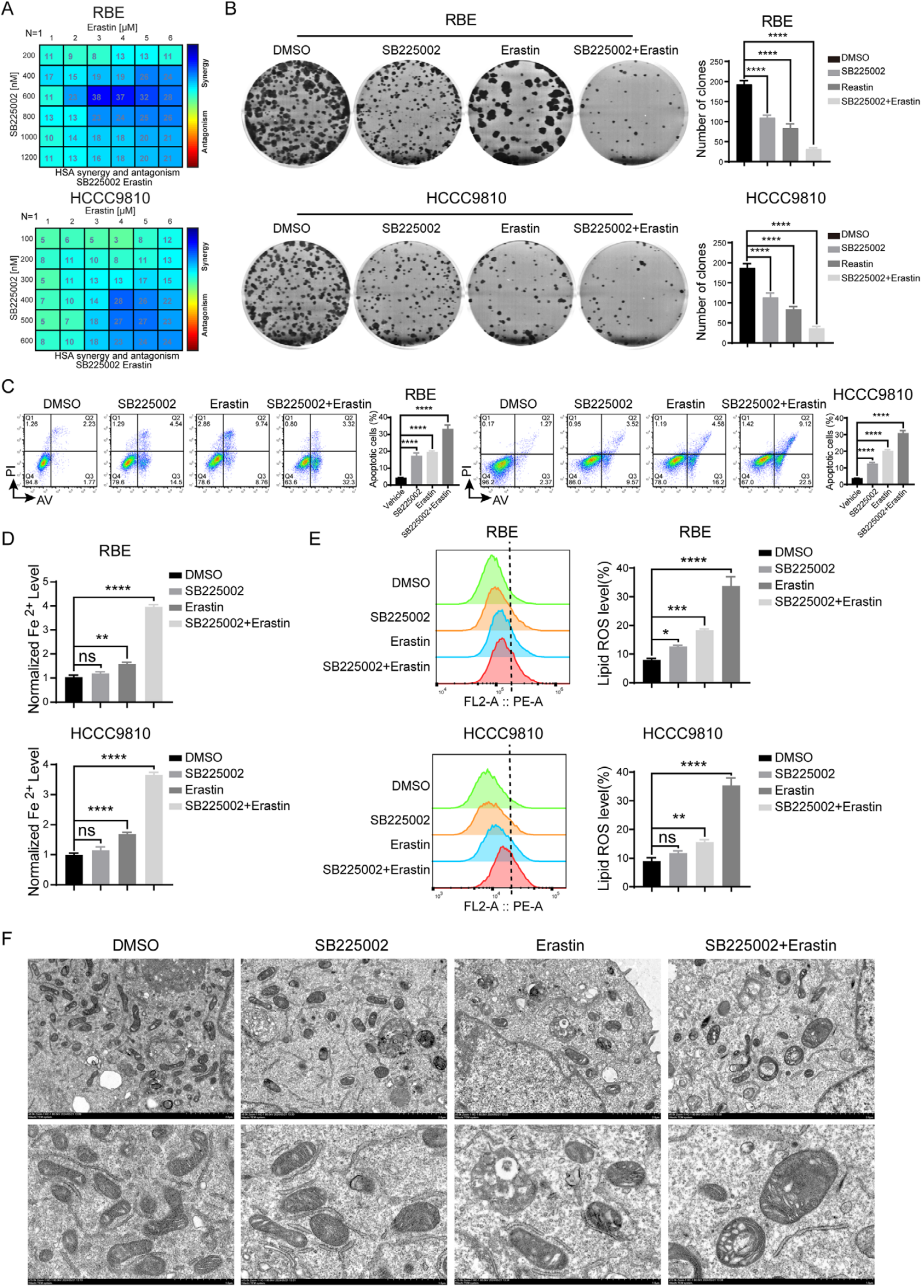
SB225002 and Erastin synergistically inhibit malignant progression of ICC cells and promote ferroptosis. (A) HCCC9810 and RBE cell lines were treated with increasing doses of Erastin combined with SB225002 for 24 h, and proliferation was monitored by CCK8‐kit. (B) Colony formation assay was performed in the HCCC9810 and RBE cells treating with DMSO, Erastin (3 µm), SB225002 (400 nm), and Erastin (3 µm) combined with SB225002(400 nm) respectively. Data are means ± S.D., n = 3. (C) Apoptosis assay was performed in the HCCC9810 and RBE cells treating with DMSO, Erastin (3 µm), SB225002 (400 nm), and Erastin (3 µm) combined with SB225002(400 nm) respectively. Data are means ± S.D., n = 3. (D) The Fe^2+^ assays in ICC cells treating with DMSO, Erastin (3 µm), SB225002 (400 nm), and Erastin (3 µm) combined with SB225002(400 nm) respectively. Data are means ± S.D., n = 3. (E) The lipid ROS assays in ICC cells treating with DMSO, Erastin (3 µm), SB225002 (400 nm), and Erastin (3 µm) combined with SB225002(400 nm) respectively. Data are means ± S.D., n = 3. (F) Transmission electron microscopy images captured following exposure to increasing doses of elesclomol for 24 h in HCCC9810 and RBE cells treated with DMSO, Erastin (3 µm), SB225002 (400 nm), and Erastin (3 µm) combined with SB225002(400 nm) respectively. Data are means ± S.D., n = 3. ^*^ *p* < 0.05, ^**^ *p* < 0.01, ^***^ *p* < 0.001, ^****^ *p* < 0.0001.

Our previous findings demonstrate that the CXCL5/CXCR2 axis plays a critical role in regulating the malignant progression and ferroptosis of ICC. Based on these results, we hypothesized that the CXCR2 inhibitor SB225002 could synergize with the ferroptosis inducer Erastin to inhibit ICC progression and enhance ferroptosis. To test this hypothesis, we conducted combination drug experiments using SB225002 and Erastin. Results revealed a significant synergistic effect when the two agents were administered together (HCCC9810: SB225002 400 nm, Erastin 4 µm; RBE: SB225002 600 nm, Erastin 3 µm) (Figure 6A and Figure S5A,B). We next assessed the impact of combination therapy vs. monotherapy on ICC proliferation and apoptosis using colony formation assays and flow cytometry, respectively. Compared to single‐agent and control groups, the combination treatment group exhibited a marked reduction in colony‐forming ability (Figure 6B) and a significant increase in apoptotic cell proportion (Figure 6C), indicating that SB225002 and Erastin synergistically inhibit ICC malignant progression. To evaluate ferroptosis induction, we measured intracellular Fe^2+^ concentrations and lipid ROS levels. The combination group demonstrated significantly higher Fe^2+^ and lipid ROS levels compared to monotherapy and control groups (Figure 6D,E). Furthermore, transmission electron microscopy (TEM) revealed pronounced mitochondrial shrinkage in the combination group, providing direct morphological evidence of enhanced ferroptosis (Figure 6F). In summary, these findings demonstrate that SB225002 and Erastin synergistically inhibit the malignant progression of ICC cells and promote ferroptosis, offering a promising therapeutic strategy for ICC treatment.

### 3.7 Synergistic Therapeutic Efficacy of SB225002 and Erastin in ICC

**FIGURE 7:**
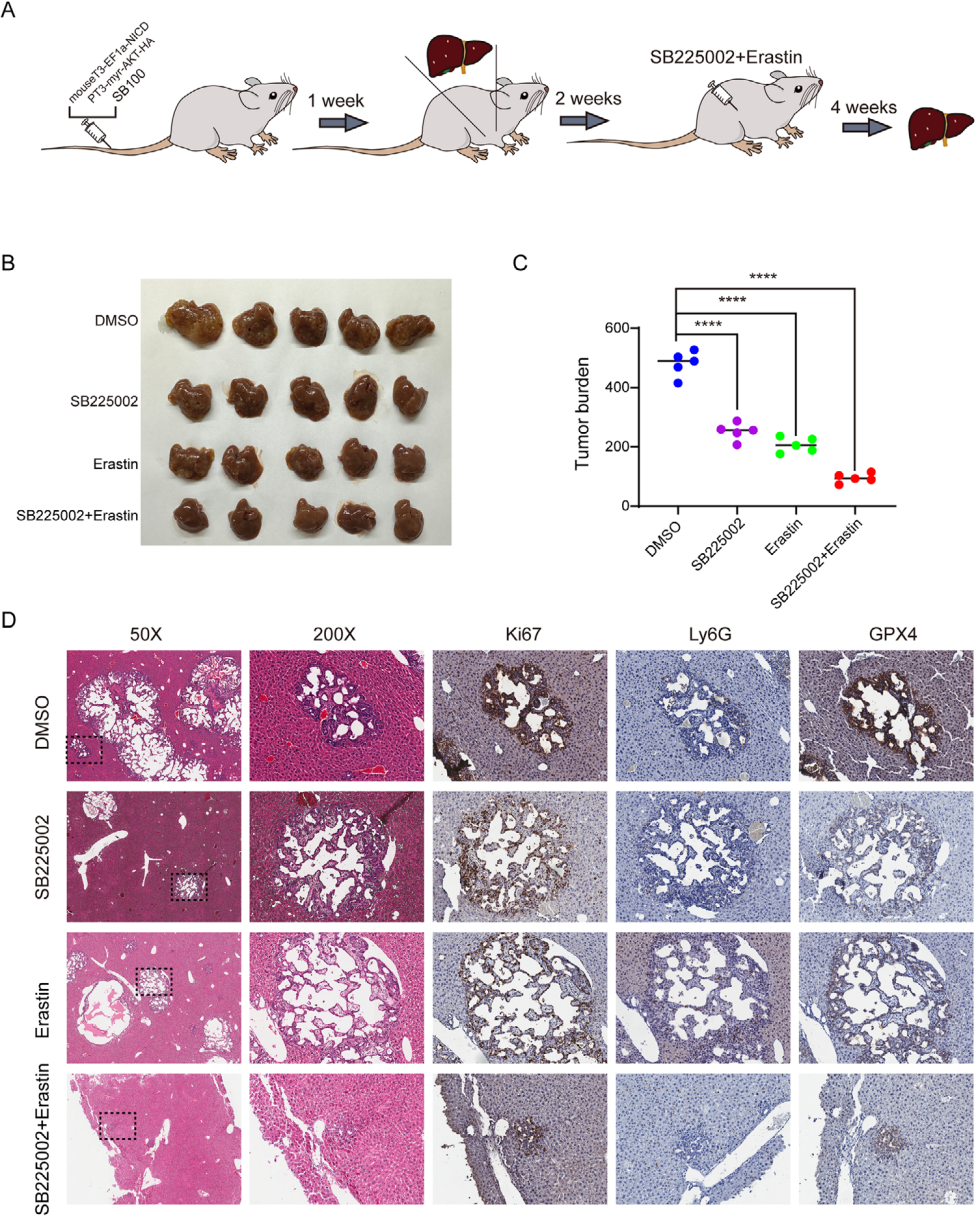
The combination of SB225002 and Erastin to treat ICC. (A) The diagram shows the procedure of the construction of ICC in situ model in mice. (B) Images of representative tumors for every group at day 28. (C) Changes of tumor burden treated with DMSO, Erastin (5 mg/kg), SB225002 (2 mg/kg), and Erastin (5 mg/kg) combined with SB225002 (2 mg/kg) respectively during day 28. (D) IHC staining using Hematoxylin and eosin (H&E) for Ki67, ly6G and GPX4. Data are means ± S.D., n = 5. ^*^ *p* < 0.05, ^**^ *p* < 0.01, ^***^ *p* < 0.001, ^****^ *p* < 0.0001.

To further validate the therapeutic potential of the combination regimen, we conducted in vivo experiments using an established ICC mouse model. Spontaneous in situ ICC was induced via hydrodynamic tail vein injection of plasmids, followed by a 4‐week treatment regimen with SB225002, Erastin, or their combination (Figure 7A). After treatment, mice were euthanized, and livers were excised for analysis. Quantitative evaluation of liver surface lesions exhibited a pronounced diminution in the combination intervention group relative to control and monotherapy arms, demonstrating enhanced therapeutic efficacy (Figure 7B,C). To further evaluate the effects of the combination therapy, hematoxylin and eosin (H&E) staining and immunohistochemical analysis (Ki67, Ly6G, GPX4) were performed. Results indicated a marked decrease in tumor nodules and neutrophil infiltration in the combination group compared to monotherapy and control groups, suggesting that the combination treatment synergistically inhibits ICC malignant progression, suppresses neutrophil recruitment, and induces ferroptosis (Figure 7D). We also calculated the overall survival of the mice after they were treated with different drugs. The results showed that the overall survival of the combined treatment group was significantly prolonged (Figure S5D). To evaluate the toxicity of drugs, H&E staining is performed on the heart, spleen, and kidney of mice treated with different drugs. The results showed no distinguishable difference between different groups (Figure S5E). Furthermore, we also established a mouse subcutaneous tumor model of ICC cells treated with SB225002, Erastin, or their combination. The results showed that combination treatment inhibit the growth of tumors better (Figure S5F,G). In summary, these findings demonstrate that the combination of SB225002 and Erastin exhibits significant synergistic efficacy in treating ICC, highlighting its potential as a promising therapeutic strategy.

### 3.8 Construction and Validation of a Prognostic Model Based on the CXCR2/CXCL5/p65/PTGS2 Axis in ICC

**FIGURE 8:**
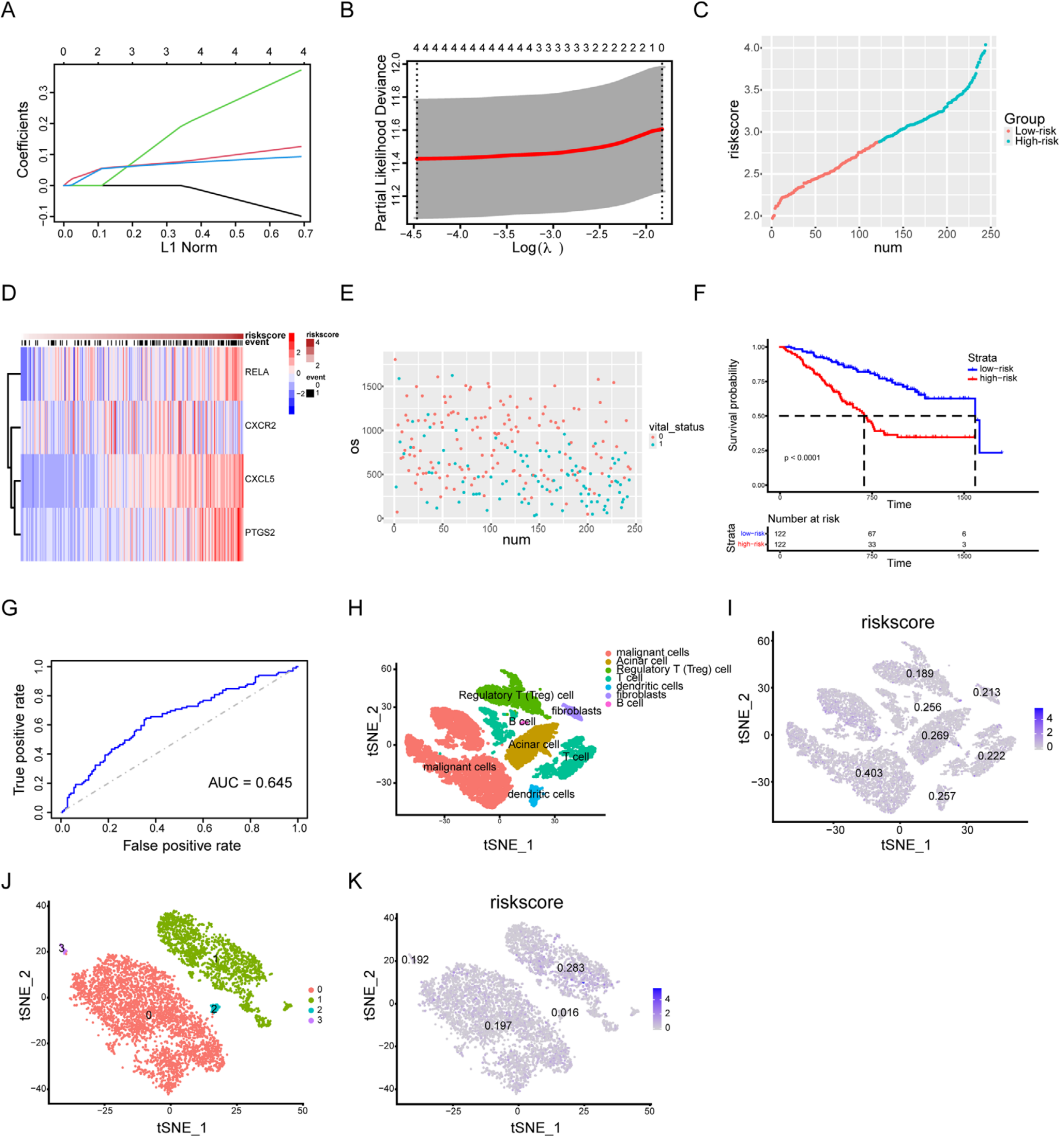
The CXCR2/CXCL5/p65/PTGS2 axis constructs an ICC prognostic model. (A) LASSO deviance profile of the 4 intersection genes. (B) LASSO regression coefficient profile of the 4 intersection genes. (C) The risk scores of ICC in NODE database (OEZ008243). Low risk score was represented by 1, high risk score was represented by 2. The “num” on the x‐axis denotes the labels of the 255 patients in the dataset, ordered from low to high risk scores. (D) Risk score in the NODE training set, patient survival, and expression of 4 genes in the training set. (E) The distribution of survival status and risk scores in ICC patients in the training set. Low risk score was represented by 0, high risk score was represented by 1. Num represented the patient's identification number. (F) Kaplan‐Meier curves of patients in high and low risk score groups for overall survival. (G) ROC curve of the risk score model in the training set. (H) The cells were categorized into 7 clusters based on different cell markers (GSE138709). (I) Scatter plots show the risk score expression distribution in different cell clusters. (J) The cells were categorized into 4 subtypes based on single‐cell RNA sequencing data. 0–3 means different subtypes. (K) Scatter plots show the risk score expression distribution in different subtypes.

Our results revealed the CXCR2/CXCL5/p65/PTGS2 axis is a critical pathway regulating ferroptosis in ICC. To leverage this pathway for clinical applications, we aimed to construct a ferroptosis‐related prognostic model. Using LASSO regression analysis on the OEZ008243 expression dataset, we developed a risk score model according to the expression of CXCR2, CXCL5, p65 (RELA), and PTGS2. The risk score was calculated as follows: Risk Score = CXCL5 × 0.115561519 + CXCR2 × (‐0.077185043) + RELA × 0.333959129 + PTGS2 × 0.089333218 (Figure 8A–C). Survival analysis and ROC curve evaluation demonstrated that patients stratified into the high‐risk category showed significantly reduced long‐term survival relative to the low‐risk group. (Figure 8D–G). To validate the model, we applied it to the GSE89749 dataset and proteomics data, which confirmed that high‐risk patients exhibited significantly worse outcomes than low‐risk patients (Figure S6A–D). To further assess the model's effectiveness and reliability at the single‐cell level, we analyzed ICC single‐cell RNA sequencing data. Results revealed that the risk score was significantly higher in clusters enriched with malignant tumor cells compared to other cell clusters (Figure 8H,I and Figure S6E). We further conducted ROC curve evaluation based on the tumor stage. The results showed good predictive effect in stage II and III ICC tumors (Figure S6F). Furthermore, we classified malignant cells into 4 subtypes and analyzed the riskscore. The results showed that risk score in subtype1 is the highest, and the risk score in subtype2 is the lowest (Figure 8J,K). In summary, we successfully constructed a ferroptosis‐related prognostic model based on the CXCR2/CXCL5/p65/PTGS2 axis and validated its predictive accuracy and reliability across bulk and single‐cell sequencing datasets.

## 4 Discussion

Ferroptosis exhibits significant antitumor potential by inhibiting tumor growth and progression [24]. However, the application of ferroptosis‐targeted therapies in ICC faces several challenges [25]. Key limitations include the insufficient selectivity and specificity of ferroptosis inducers, which may result in off‐target toxicity to normal cells. Additionally, tumor cells can develop resistance to ferroptosis by upregulating antioxidant mechanisms, thereby compromising therapeutic efficacy. The intricate composition of the tumor niche additionally modulates ferroptotic susceptibility, potentially limiting its effectiveness [26]. Given these challenges, there is a pressing need to explore innovative approaches to enhance the efficiency and specificity of ferroptosis‐inducing agents in cancer treatment. Our findings demonstrate that inhibition of the CXCL5/CXCR2 axis enhances the sensitivity of ICC cells to ferroptosis inducers. This study provides a critical theoretical foundation for targeting immune pathways to suppress ferroptosis resistance.

Within the tumor microenvironment, the CXCL5/CXCR2 axis exhibits dual functionality: it supports tumor growth, angiogenesis, and metastasis by promoting the recruitment of tumor‐associated neutrophils (TANs) and macrophages, while in certain contexts, it may enhance antitumor immunity by increasing the activity and infiltration of antitumor immune cells [27]. Studies have demonstrated that CXCL5 is highly expressed in cholangiocarcinoma, where it promotes tumor invasion and migration through autocrine signaling. Inhibition of CXCR2 significantly attenuates these effects [21]. Furthermore, the CXCL5/CXCR2 axis recruits neutrophils into the TME, where they release pro‐inflammatory and pro‐tumor factors that enhance tumor cell invasion and migration, thereby facilitating ICC metastasis and recurrence [28, 29]. Our findings reveal that the CXCL5/CXCR2 axis recruits N2 TANs to inhibit ferroptosis in ICC, uncovering a novel immune cell‐dependent regulatory mechanism of ferroptosis. This study presents a critical theoretical basis for combined targeting of immune pathways and RCD in ICC treatment, offering new insights into therapeutic strategies that integrate immunity and ferroptosis regulation.

PTGS2, also known as cyclooxygenase‐2 (COX‐2), exerts various cellular functions, including inflammation, pain, fever, vasodilation, and platelet aggregation [30]. In the context of tumorigenesis and cancer progression, PTGS2 contributes to tumor cell proliferation, angiogenesis, invasion, and metastasis by fostering an immunosuppressive and pro‐tumor inflammatory microenvironment [31]. Recently, the association between PTGS2 and ferroptotic pathways has gained considerable attention in scientific investigations. PTGS2 expression is markedly upregulated during ferroptosis and serves as a key biomarker of this process [32]. PTGS2 is involved in the metabolic regulation of lipid peroxidation, a central event in ferroptosis, and is closely associated with dysregulation of the cellular antioxidant system [33]. Our study revealed that the CXCL5/CXCR2 axis activates the transcriptional regulation of PTGS2. Previous research has identified NF‐κB as a critical transcription factor for PTGS2 [34]. Through promoter activity assays and ChIP experiments, we demonstrated that the CXCL5/CXCR2 axis transcriptionally activates PTGS2 via NF‐κB phosphorylation. These findings establish a mechanistic link between the CXCL5/CXCR2 axis and RCD through the inflammation‐related transcription factor NF‐κB and the PTGS2 protein. This connection provides novel potential targets for the diagnosis and treatment of ICC by targeting RCD pathways.

Our preliminary studies revealed that the CXCL5/CXCR2 axis suppresses ICC ferroptosis through two distinct mechanisms: N2 TANs recruitment and PTGS2 upregulation. However, potential synergistic interactions between these pathways remain unclear. Previous studies have demonstrated that neutrophils can activate the NF‐κB signaling pathway in tumor cells via TNF‐α [35]. Concurrently, our preliminary investigations using dual‐luciferase reporter assays and ChIP assays confirmed that the CXCL5/CXCR2 axis transcriptionally activates PTGS2 by promoting NF‐κB phosphorylation. Recent research indicates that PTGS2 enhances neutrophil chemotactic capacity through CCL2 and CCL7 secretion [36], though its precise mechanistic role in ICC requires further validation. Collectively, we hypothesize that neutrophils may activate PTGS2 expression via the NF‐κB pathway, while PTGS2 reciprocally enhances N2 TANs recruitment through CCL2/CCL7‐mediated chemotaxis, thereby establishing a positive feedback regulatory loop that synergistically inhibits ferroptosis in ICC cells. Through ELISA and chemotaxis assays, this study demonstrates that N2 TANs upregulate PTGS2 via NF‐κB pathway activation while promoting N2 TANs chemotaxis through CCL2/CCL7 secretion. These mechanistic insights suggest potential therapeutic synergy between CXCR2 inhibitors and ferroptosis inducers. Our findings provide both theoretical foundation and strategic framework for developing combinatorial therapeutic approaches targeting ICC.

Our evidence suggests that immune‐related genes may play a regulatory role in the induction of ferroptosis [37, 38]. In our study, we demonstrated through ROS assays that inhibition of the CXCL5/CXCR2 axis enhances ferroptosis in ICC. CXCR2 inhibitors, such as SB225002, specifically target the CXCR2 receptor to suppress tumor growth, metastasis, and angiogenesis, showing therapeutic potential in various cancers, including ICC [39]. Building on these findings, we hypothesized that combining a CXCR2 inhibitor with a ferroptosis inducer could synergistically enhance the therapeutic efficacy against ICC. Through systematic investigation and experimental validation, we identified that the combination of SB225002 and Erastin exhibits significant synergistic effects in treating ICC. Our study not only provides a theoretical foundation for targeting ferroptosis in ICC but also proposes a promising combination drug strategy, offering new insights into the development of effective therapeutic interventions for this aggressive malignancy.

The in vivo model used in this study may not fully recapitulate the complex tumor microenvironment and heterogeneity of human ICC. Thus, subsequent validation of the mechanisms and combination therapy regimen is needed in more physiologically relevant models. Furthermore, the prognostic model constructed in this study was not compared with standard clinicopathological variables. However, compared with existing ferroptosis‐related prognostic models, the model trained in this study exhibited higher AUC values, particularly in patients with stage II and III ICC.

## 5 Conclusions

In this study, we discovered that inhibition of the immune‐related CXCL5/CXCR2 axis significantly increases the sensitivity of ICC to ferroptosis inducers. Mechanistically, the CXCL5/CXCR2 axis suppresses ferroptosis in ICC through two distinct pathways: by phosphorylating NF‐κB and transcriptionally activating PTGS2, and by recruiting N2 TANs, which further inhibit ferroptosis. Single‐cell sequencing analysis confirmed the feasibility of the CXCR2/CXCL5/p65/PTGS2 axis as a robust molecular marker for ICC prognosis. Notably, we identified a synergistic therapeutic effect between the CXCR2 inhibitor SB225002 and the ferroptosis inducer Erastin, offering a novel combination strategy for ICC treatment. Our findings, rooted in the exploration of immune‐related genes, provide a comprehensive theoretical foundation and a promising therapeutic approach for targeting ferroptosis in ICC, paving the way for more effective treatment strategies.

## Author Contributions

Conceptualization: JR, QL, QJ, and KFL; Methodology: CQY, SYY, YZ, QJ, KFL, WLG and YQH; Investigation: CQY, SYY, YZ, QJ, WLG, RYC, and WGF; Visualization: CQY, KFL, SYY, YZ, WGF, and MHW; Supervision: YQH, RYC, WGF, QL, ZJY, and YXM; Software: WLG, YT, JQC, and YD; Writing – original draft: CQY, SYY, YZ, QJ, and RB; Writing – Review & Editing: JR, QL, KFL, and QJ; Funding acquisition: JR, JQC, and YD. All authors have read and approve the final version of the manuscript.

## Ethics Statement

All procedures involving the care and use of animals in the study were reviewed and approved by the Institutional Animal Care and Use Committee (IACUC) of ZJCLA. And the tumor sample collection protocol was approved by the Institutional Animal Care and Use Committee (IACUC) of ZJCLA (protocol number: ZJCLA‐IACUC‐20010826). Whole blood samples in the study obtained written informed consent from the volunteers and was approved by the Clinical Research Ethics Committee of the First Affiliated Hospital, Zhejiang University (approve number: [2026B] IIT Ethics Approval No. 0571).

## Conflicts of Interest

The authors declare no conflicts of interest.

## Supporting information




**Supporting File 1**: advs77185‐sup‐0001‐SuppMat.doc.

**Supporting File 2**: advs77185‐sup‐0002‐SuppMat.xlsx.

## Data Availability

The data that support the findings of this study are available on request from the corresponding author. The data are not publicly available due to privacy or ethical restrictions.
