# Supplementary material for: The CXCL5/CXCR2 Axis Attenuates Ferroptosis in Intrahepatic Cholangiocarcinoma Through the Positive Feedback Loop Between PTGS2 Transcriptional Activation and Neutrophil Recruitment
Source: Adv Sci (Weinh). 2026 Aug 17:e77185. Online ahead of print. doi: 10.1002/advs.77185 (PMC13479846; doi:10.1002/advs.77185)
Supplement: Supplementary file 1 [file ADVS-9999-e77185-s002.doc]

**Supplementary Materials**

**The CXCL5/CXCR2 Axis Attenuates Ferroptosis in Intrahepatic Cholangiocarcinoma through the Positive Feedback Loop between PTGS2 Transcriptional Activation and Neutrophil Recruitment**

**Chanqi Ye^1*^, Kefeng Lin^2*#^, Shuyao Yang^3*^, Yan Zhang^2*^, Wenli Gao^4*^,** **Jiaqi Chen^2^, Ying Dong^2^, Rui Bai^2^, Yang Tian^5^, Minghao Wang^2^, Zhijun Yuan^6^, YuXiao Ma^7^, Yuqin He^1^, Ruyin Chen^1^, Wenguang Fu^8^, Qi Jiang^1#^, Qiong Li^1#^, Jian Ruan^1,8#^**

^1^ Department of Medical Oncology, The First Affiliated Hospital, Zhejiang University, School of Medicine, and Key Laboratory of Cancer Prevention and Intervention, Ministry of Education, Hangzhou 310003, Zhejiang Province, People’s Republic of China.

^2^ Department of Medical Oncology, Cancer Institute, Key Laboratory of Cancer Prevention and Intervention, Ministry of Education, The Second Affiliated Hospital of Zhejiang University, School of Medicine, Hangzhou, Zhejiang 310009, China.

^3^ Department of Medical Oncology, Nanyang Second General Hospital, Nanyang, Henan 473000, China

^4^ Department of Laboratory Medicine, Institute of Traditional Chinese Medicine of Sichuan Academy of Chinese Medicine Sciences, Chengdu, Sichuan, China.

^5^ Department of Hepatobiliary and Pancreatic Surgery, The Second Affiliated Hospital, Zhejiang University School of Medicine, Hangzhou, 310009, China.

^6^ Department of Pathology and Pathophysiology, Zhejiang University School of Medicine, Hangzhou, Zhejiang, China

^7^ Department of General Surgery, Shizhu Hospital of traditional Chinese Medicine, Shizhu County, Chongqing, 409100, China

^8^ Department of Hepatobiliary Surgery, The Affiliated Hospital of Southwest Medical University, Luzhou 646000, Sichuan Province, People’s Republic of China.

^*^ These authors contributed equally to this work.

^#^ Correspondence:

Jian Ruan, Email: [software233@zju.edu.cn](mailto:software233@zju.edu.cn)

Qiong Li, Email: [liqiong0513@zju.edu.cn](mailto:liqiong0513@zju.edu.cn)

Kefeng Lin, Email: [kd88@zju.edu](mailto:liqiong0513@zju.edu).cn

Qi Jiang, Email: 1515014@zju.edu.cn

**Figure S1 CXCL5/CXCR2 axis regulates the ferroptosis and malignant progression of ICC**

(A) The IC50 values of RBE and HCCC9810 treating with Erastin and RSL3 in shNC or shCXCL5/CXCL3 groups. Data are means ± S.D., n=3. (B) Western blot analysis of CXCL5 protein expressions in ICC cell lines and human intrahepatic bile duct epithelial cells (HIBEC). (C) Volcano plots illustrate differentially regulated genes expression between CXCL5 high expression and CXCL5 low expression in NODE database (OEZ008243). The groups were stratifed according to the median expression level of CXCL5. Values are presented as the log2 of foldchange. (D) The Fe^2+^ assays in HCCC9810 and RBE cell lines treating with low concentrations of Erastin (2 µM) and RSL3 (1 µM) when CXCL5 was knockdown. Data are means ± S.D., n=3. (E) The lipid ROS assays in HCCC9810 and RBE cell lines treating with low concentrations of Erastin (2 µM) and RSL3 (1 µM) in CXCL5 knockdown group. Data are means ± S.D., n=3. (F) Colony formation assays in the HCCC9810 and RBE cell lines when knocking down CXCL5. Data are means ± S.D., n=3. (G) The apoptosis assays in the HCCC9810 and RBE cell lines treated with low concentrations of Erastin (2 µM) when knocking down CXCL5. Data are means ± S.D., n=3. (H) Western blot analysis of CXCL5, BAX, and BCL2 protein expressions in ICC cell line that with or without Erastin (2 µM) treatment after CXCL5 knockout. *P < 0.05, ** P < 0.01, *** P < 0.001, **** P < 0.0001.

**Figure S2 CXCL5/CXCR2 axis regulates ICC ferroptosis through PTGS2**

(A) The IC50 values of HCCC9810 treating with Erastin and RSL3 in shNC group. (B) The IC50 values of HCCC9810 treating with Erastin and RSL3 in shPTGS2 group. Data are means ± S.D., n=3. (C) The Fe^2+^ assays in HCCC9810 and RBE cell lines treating with low concentrations of Erastin (2 µM) and RSL3 (1 µM) when knock down PTGS2. Data are means ± S.D., n=3. (D) The lipid ROS assays in HCCC9810 and RBE cell lines treating with low concentrations of Erastin (2 µM) and RSL3 (1 µM) when knock down PTGS2. Data are means ± S.D., n=3. (E) The GSH assays in HCCC9810 and RBE cell lines treating with low concentrations of Erastin (2 µM) and RSL3 (1 µM) when knock down PTGS2. Data are means ± S.D., n=3. (F) The Fe^2+^ assays in knocking down CLXC5 and overexpressing PTGS2 ICC cell lines when treating with low concentrations of Erastin (2 µM) and RSL3 (1 µM). Data are means ± S.D., n=3. (G) The lipid ROS assays in knocking down CLXC5 and overexpressing PTGS2 ICC cell lines when treating with low concentrations of Erastin (2 µM) and RSL3 (1 µM). Data are means ± S.D., n=3. *P < 0.05, ** P < 0.01, *** P < 0.001, **** P < 0.0001.

**Figure S3 The potential binding sites of PTGS2 targeted by p65**

The predictive result of the binding sites of PTGS2 targeted by p65 in PROMO database.

**Figure S4 CXCL5 promotes malignant progression of ICC and inhibits ferroptosis by recruiting N2 TANs**

(A) The boxplot shows the distribution of different immune cells between CXCL5 high and low expression groups in OEZ008243. The high and low groups were divided according to the median expression of CXCL5. (B) Western blot analysis of ARG1, MMP9, PD-L1 protein expressions in neutrophils isolated from human whole blood and the induced N2 TAN. P1 to P5 represent different volunteers. (C) TNF-α concentration in medium of neutrophils and N2 TANs co-cultured with ICC cell respectively was measured by ELISA. *P < 0.05, ** P < 0.01, *** P < 0.001, **** P < 0.0001.

**Figure S5 The combination of SB225002 and Erastin to treat ICC**

(A) The inhibition effect percentage of Erastin combined with SB225002 with the increasing doses. (B) IC50 values of Erastin and SB225002 in HCCC9810 cells. (C) IC50 values of Erastin and SB225002 in RBE cells. (D) Overall survival of mice treated with SB225002, Erastin, and combination of them. Data are means ± S.D., n=5. (E) H&E-stained images of heart, spleen, and kidney of mice treated with SB225002, Erastin, and combination of them. Data are means ± S.D., n=5. (F) Images of tumors for every group. Data are means ± S.D., n=5. (G) Weight of tumors dissected from mice of different groups. Data are means ± S.D., n=5. *P < 0.05, ** P < 0.01, *** P < 0.001, **** P < 0.0001.

**Figure S6 The LASSO model in the validation set**

(A) Risk score in the NODE test set, patient survival, and expression of 4 genes in the test set (GSE89749). (B) The distribution of survival status and risk scores in ICC patients in the test set. Low risk score was represented by 0, high risk score was represented by 1. (C) Kaplan-Meier curves of patients in high and low risk score groups for overall survival (left: GSE89749 and right: proteomics data). (D) ROC curve of the risk score model in the test set (left: GSE89749 and right: proteomics data). (E) The distributions of signature genes in different cell populations in ICC. (F) ROC curve of the risk score model in different tumor stages in the GES89749.
